# Comparative analysis of proteome maps of silkworm hemolymph during different developmental stages

**DOI:** 10.1186/1477-5956-8-45

**Published:** 2010-09-08

**Authors:** Yong Hou, Yong Zou, Fei Wang, Jing Gong, Xiaowu Zhong, Qingyou Xia, Ping Zhao

**Affiliations:** 1 College of Biotechnology, Institute of Sericulture and Systems Biology, Southwest University, Chongqing 400716, PR China

## Abstract

**Background:**

The silkworm *Bombyx mori *is a lepidopteran insect with four developmental stages: egg, larva (caterpillar), pupa, and adult. The hemolymph of the silkworm is in an open system that circulates among all organs, and functions in nutrient and hormone transport, injury, and immunity. To understand the intricate developmental mechanisms of metamorphosis, silkworm hemolymph from different developmental stages, including the 3^rd ^day of fifth instar, the 6^th ^day of fifth instar, the 3^rd ^day of pupation, the 8^th ^day of pupal stage and the first day of the moth stage, was investigated by two-dimensional electrophoresis and mass spectrometry.

**Results:**

Two-dimensional polyacrylamide gel electrophoresis showed that from the larval to moth stages, silkworm hemolymph proteins changed markedly. Not only did major proteins such as SP1, SP2, and the 30 K lipoprotein change, but other proteins varied greatly at different stages. To understand the functions of these proteins in silkworm development, 56 spots were excised from gels for analysis by matrix-assisted laser desorption ionization time-of-flight mass spectrometry (MALDI-TOF MS). We identified 34 proteins involved in metamorphosis, programmed cell death, food digestion, metabolism, and nutrient storage and transport. Most proteins showed different expression at different stages, suggesting functions in development and metamorphosis. An abundance of proteins related to immunity were found, including hemolin, prophenoloxidase, serine proteinase-like protein, paralytic peptide-binding protein, and protease inhibitor.

**Conclusions:**

Proteomics research not only provides the opportunity for direct investigation of protein expression patterns, but also identifies many attractive candidates for further study. Two-dimensional maps of hemolymph proteins expressed during the growth and metamorphosis of the silkworm offer important insights into hemolymph function and insect metamorphosis.

## Background

The mulberry silkworm, *Bombyx mori*, has been raised for more than 5000 years in Asian countries, and is a major economic resource for many families. Currently, the silkworm is not only a domesticated insect used for silk production, but is also a model lepidopteran for pest control studies. The silkworm has an open circulatory system containing hemolymph, which surrounds the tissues of the silkworm with blood. Nutrients and oxygen are delivered to all parts of the silkworm body through the hemolymph, which is also an important depository for nutrition and energy. After eating, the percentage of hemolymph in the silkworm increases as a proportion of body weight, while a silkworm that is starved or has just finished ecdysis has a reduced proportion of hemolymph. In addition, hemolymph has a key role in innate immunity response, that is triggered when bacteria or fungi enter the silkworm body [1].

Since the 1970 s and earlier, the proteins of silkworm hemolymph have been studied to elucidate their role in silkworm development. In 1953, Telfer identified vitellogenin, a female-specific protein in the hemolymph of *Hyalophora cecropia*, as the first vitellogenin found in insects [2]. In 1980 s, two major proteins, SP1 (storage protein 1) and SP2 (storage protein 2), were discovered in silkworm larval hemolymph. These two proteins show clear variability and sex-differentiation during last instar stage development [3,4]. In 1981, a group of structurally related proteins called 30 K proteins because of their approximate molecular weights of 30,000 Da, were found to be stored in the larval hemolymph of silkworms in a stage-dependent fashion. The 30 K proteins are minimally detectable in the hemolymph before day 3 of the fifth larval instar, but become major hemolymph proteins in the early pupal stage because of progressive increase in expression after the 3^rd ^day of fifth instar larvae [5]. After genome maps of the silkworm were published in 2004 and 2008 [6,7], proteomic technology was applied to silkworm research. Proteomic tools, particularly two-dimensional (2D)-electrophoresis in conjunction with mass spectrometry have been used to analyze silkglands, fatbody, skeletal muscle, and hemolymph proteins [8-11]. In this work, hemolymph proteins from different developmental stages were investigated by 2D-electrophoresis and matrix-assisted laser desorption ionization time-of-flight mass spectrometry (MALDI-TOF MS), to construct a profile of silkworm hemolymph proteins from larva to moth, to aid in a comprehensive understanding of silkworm metamorphosis.

## Results

### SDS-PAGE of silkworm hemolymph protein from 1^st ^day of fifth instar to moth

**Figure 1:**
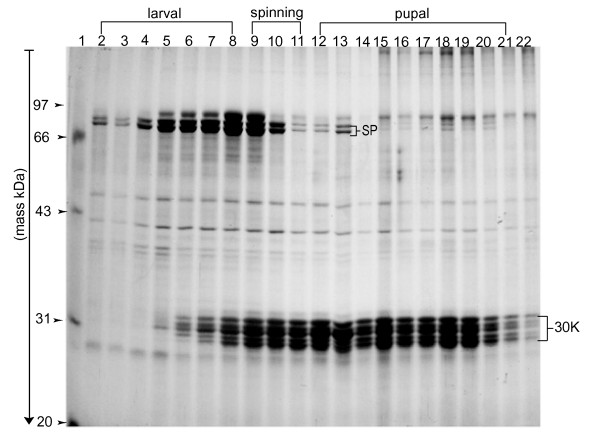
**SDS-PAGE analysis of silkworm hemolymph from the 1^st ^day of fifth instar to moth**. Hemolymph (15 μg) protein was applied to each lane and separated by 12% resolving gel. SDS-PAGE was carried out at 10 mA for stacking gel and 20 mA for resolving gel. Gels were stained with silver nitrate according to the Amersham 2D handbook. 1: Low molecular markers; 2-8: 1^st ^day to 7^th ^day in last larval stage; 9-11: 1^st ^day to 3^rd ^day in spinning stage; 12-21: 1^st ^day to 10^th ^day in pupal stage; 22: eclosion.

Silkworm hemolymph from the 1^st ^day of fifth instar to eclosion was collected and analyzed by SDS-PAGE (Fig. 1). During this period, silkworms underwent two dramatic morphological transitions, pupation and eclosion. The SDS-PAGE map showed that hemolymph proteins, particularly the proteins around 30 kDa and 80 kDa, underwent dramatic changes. Proteins around 80 kDa were detected at the 1^st ^day of fifth instar, and their expression level gradually increased from the 3^rd ^day of fifth instar, reaching a maximum at the wandering stage, then descending acutely, and almost disappearing during pupation. Proteins of approximately 30 kDa appeared in the hemolymph at the 4^th ^day of fifth instar, increased gradually as the silkworm grew, and became a major component of hemolymph in pupation. At the late pupa stage, proteins around 30 kDa began to decrease, almost disappearing during eclosion. By molecular weight and expression patterns, these proteins were predicted to be storage proteins and 30 K proteins of silkworm hemolymph [12]. They were expressed in the fatbody and secreted to the hemolymph, and constituted the major part of plasma proteins. Considering the limitation of SDS-PAGE resolution, a few samples were selected for further analysis by 2D-electrophoresis.

### Two-dimensional electrophoresis of silkworm hemolymph at different stages

**Figure 2:**
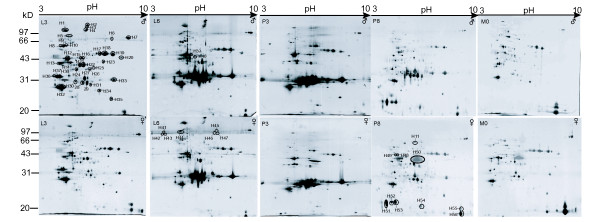
**Two-dimensional electrophoresis of silkworm hemolymph at different stages**. Male(♂) and female(♀) hemolymph sample were subjected to 2D-electrophoresis separately. Protein (60 μg) was separated by IEF using 13 cm IPG strips (pH 3-10), followed by 12.5% SDS-PAGE and silver-staining. L3: 3^rd ^day fifth instar larva; L6: 6^th ^day fifth instar larval; P3: 3^rd ^day pupa; P8: 8^th ^day pupa; M0: eclosion. Circles indicate 56 spots excised for MALDI-TOF MS, most of which had different expression during development and metamorphosis.

Samples from the 3^rd ^day of fifth instar (L3), the 6^th ^day of fifth instar (L6), the 3^rd ^day of pupation, the 8^th ^day of pupa, and eclosion were selected for analysis by 2D-electrophoresis. Total proteins from each sample were separated by 2D-electrophoresis at pH 3-10 (Fig. 2). The results show that approximately 128 spots proteins were observed from the L3 map of the male silkworm. The proteins ranged from 20 to 97 kDa in molecular mass, and from pH 4 to 10 in pI. Similar patterns were displayed on three replicate gels, with an average matching rate of more than 96% between replicates. From L3, proteins in the range of 28-31 kDa molecular mass were up-regulated sharply, so that separation at L6 and P3 was difficult to map. Vertical stripes appeared because of the large quantity of proteins in the 30 kDa region. In the 2D hemolymph map from the 8^th ^day of pupation and in moths, these stripes were well separated because of decreased expression of the proteins around 30 kDa. Some sex-specific proteins were observed in female, but not male silkworm hemolymph, such as spot H50 at 42 kDa. The change in proteins by 2D-PAGE was consistent with the SDS-PAGE analysis. The high resolution of 2D-electrophoresis made it possible to study gel-separated proteins by MALDI-TOF MS.

### Identification of excised spots from the hemolymph 2D map

**Figure 3:**
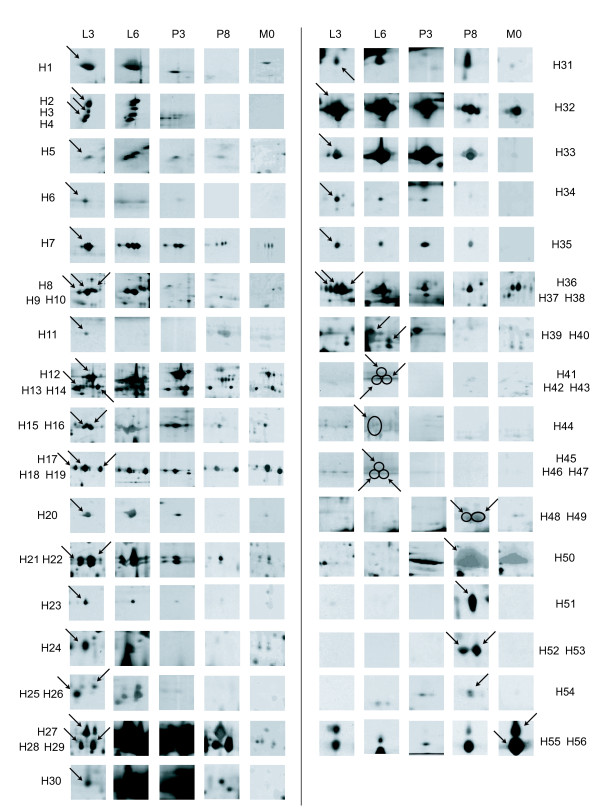
**Partial maps of silkworm hemolymph proteins from larva to moth**. L3: 3^rd ^day fifth instar larvae; L6: 6^th ^day fifth instar larvae; P3: 3^rd ^day pupa; P8: 8^th ^day pupa; M0: eclosion. More than 20 spots were expressed at the highest level in larvae, H1, H2, H3, H4, H5, H6, H7, H8, H9, H10, H20, H21, H22, H23, H24, H25, H26, H40, H41-47; 11 spots were highest in pupa, H11, H27, H28, H29, H33, H48, H49, H50, H51, H52, H53, and only two spots were highest in moth, H55, H56.

To understand the change in silkworm hemolymph proteins, 56 spots were excised for analysis by MALDI-TOF MS. The results identified by peptide mass fingerprinting are in Additional File 1. To clarify the change in hemolymph proteins during development and metamorphosis, the proteins were displayed by partial maps (Fig. 3). The results showed more than 20 spots expressed at their highest level during the larval stage, including H1, H2, H3, H4, H5, H6, H7, H8, H9, H10, H20, H21, H22, H23, H24, H25, H26, H40, and H41-47. Eleven spots had the highest level in pupa: H11, H27, H28, H29, H33, H48, H49, H50, H51, H52 and H53, and only few spots (H55, H56) were seen in the moth stage. Some proteins displayed consistent expression from the 5^th ^instar larval stage to the moth stage, such as H17, H18, H19. Among these proteins, some have been reported previously, such as vitellogenin (H50), while others were first identified in this work, such as H20, H23, and H25.

**Figure 4:**
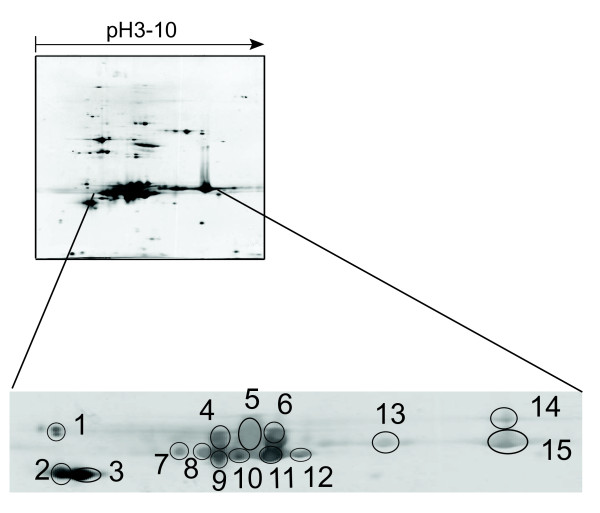
**Identification of 30 K proteins in silkworm hemolymph from 3^rd ^day of pupation by MALDI-TOF MS**. The enlarged part is the area from 27 kD-31 kD of 2D gel, where 15 spots indicated by circles were excised to be analyzed by MALDI-TOF MS spectrometry.

**Figure 5:**
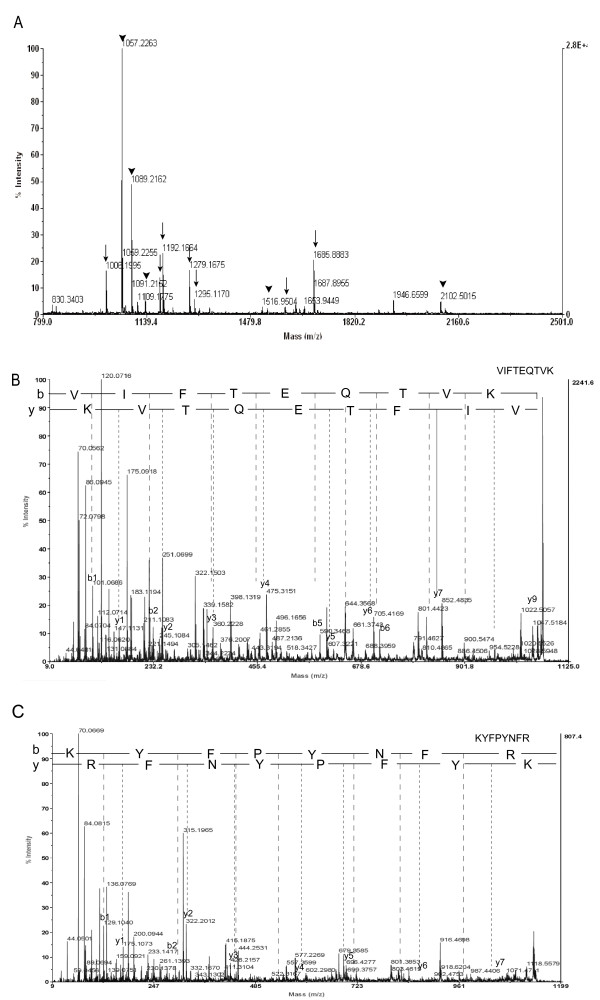
**Identification of spot 8 from 3^rd ^day of pupation by peptide mass fingerprint and MS/MS**. Spot 8 was excised from gels and digested with trypsin, and the resulting peptides were analyzed using MALDI-TOF mass spectrometry and TOF/TOF mass spectrometer. (A) MALDI-TOF MS on in-gel-digested peptides of spot 8. A total of 22 deisotoped peptide masses were submitted to a database search, and 14 peptides matched two members of the 30 K protein family of silkworm. Arrows indicate peptides from Bmlp4. Arrowheads indicate peptides from Bmlp1. (B) MS/MS spectra of ion 1064.28 from Bmlp1. (C) MS/MS spectra of ion 1134.25 from Bmlp4. Fragment ion segments from the precursor ions confirmed the results of peptide mass fingerprint. The y and b ions and their corresponding peptide sequences are shown.

To make a profile of silkworm hemolymph 30 K proteins, 15 additional spots were cut from this area for MALDI-TOF MS (Fig. 4). Five 30 K proteins were obtained from the 3^rd ^day pupa hemolymph. Because of the abundance of 30 K proteins in hemolymph, a silver-stained spot on a 2D-gel may contain several overlapping proteins. For example, both of Bmlp1 and Bmlp4 were present in spot 8, and could be identified clearly by their specific peptide mass (Fig. 5). Some proteins, however, were constitutively detected in multiple spots. For example, Bmlp7 was identified from spots 13, 14 and 15; and Bmlp1 was identified in spots 6, 11 and 12 (Additional File 2). Isoforms of proteins with unique pI values might occur because of post-translation modifications, such as phosphorylation. To validate the identification from peptide mass fingerprint, some peptides were analyzed by MS/MS. Fragment ion segments from the precursor ions confirmed the results of peptide mass fingerprint (Fig. 5).

## Discussion

In 2009, Kajiwara investigated the proteome of silkworm hemolymph by 2D-mapping and ion-trap mass spectrometry, and identified 67 proteins in a hemolymph map of the 3^rd ^day of fifth instar [13]. After comparing the 56 proteins identified in this work with those 67 proteins, we found only 5 that were identical, including juvenile hormone-binding proteins (spots H36-38); aldose reductase (spots H21, H22); low molecular-weight lipid proteins (spots H27, H28 and H29); and CI8 (spot H12). Other proteins identified by Kajiwara, such as RNA-binding proteins, carboxylesterases, zinc-finger proteins, and some hypothetical proteins, were not identified in this work. Comparing the hemolymph map of Kajiwara with the hemolymph map from this work, we found the pH range of the two maps was different. We used pH 3-10 strips to separate proteins, while Kajiwara used pH 4-7 strips. For this reason, some alkaline proteins in this work may not appear in the Kajiwara map, such as imaginal disk growth factor (spot H17-19), gelsolin (spot H20), and glyoxylate reductase (spot H23). We also compared our 2D map with the 2D map of *Manduca *hemolymph reported by Furusawa in 2007 [14], and found 10 *Manduca *hemolymph proteins had similar expression patterns in silkworm hemolymph protein, including transferrin protein, serine proteinase-like protein, chymotrypsin inhibitor, hemolin and prophenoloxidase. Thus, our results contribute to the understanding of development and metamorphosis not only in the silkworm, but also in other lepidopteran pests.

### 30 K protein of silkworm hemolymph

30 K proteins, a group of lipoproteins with a molecular mass of approximately 30 kDa, accumulate in a stage-dependent fashion in the larval hemolymph of the silkworm, *Bombyx mori*. In previous work, we analyzed the silkworm genome and expressed sequences, and found 10 genes encoding 30 K proteins. By semi-quantitative reverse-transcription polymerase chain reaction, eight members were found to be expressed in the fatbody [15]. In this work, we investigated the 30 K proteins of hemolymph at the protein level using 2D-PAGE and MALDI-TOF MS. Expression of hemolymph 30 K proteins demonstrated a dynamic change from the 1^st ^day of fifth instar larvae through eclosion, with maximum expression at the spinning stage, and almost disappearance by the 1^st ^day of moth stage. Five 30 K protein family members were identified from the 28-31 kDa area in the hemolymph of 3^rd ^day pupa (Additional File 2). The proteins sequences of Bmlp1, Bmlp2, Bmlp3 and Bmlp4 had more than 97% similarity with PBMHP-6, PBMHP-12, PBMHPC-19 and PBMHPC-21, low molecular lipoprotein 30 K of silkworm which were reported previously by Sakai [16]. Because of the high similarity, these might be the same proteins in silkworm, which show some genetic variation in different varieties. Bmlp7 is a novel 30 K protein in hemolymph, and shared 93% protein sequence similarity with PBMHPC-19. Other members were not detected in hemolymph because of low expression, or expression during other stages.

In the hemolymph map of the 3^rd ^day of fifth instar, Bmlp1, Bmlp2, Bmlp3 and Bmlp7 were found by MS, but not Bmlp4. Bmlp4 increased sharply during development, and was detected in six spots (spot 1, 4, 7, 8, 9, 10) in the hemolymph map of the 3^rd ^day of pupation. After metamorphosis from silkworm to pupa, it decreased rapidly in the hemolymph, and was not detected in the hemolymph at the 8^th ^day of pupation. Compared to Bmlp1, Bmlp2, Bmlp3 and Bmlp7, Bmlp4 is a special 30 K protein that appears later in the fifth instar stage, is expressed strongly, and disappears rapidly. This is similar to the mRNA expression of Bmlp4 in fatbody that was reported by Sun in 2007 [15]. These results suggested that Bmlp4 may have a different function than other silkworm 30 K proteins.

During the later pupal stages, female silkworm proteins around 30 kD were reduced compared to males. It has been reported that during this period, the 30 K proteins were transported into the embryo in female silkworms, and became important for embryonic development [17,18], which would result in the reduction of 30 kD in female hemolymph. While in male silkworms, the 30 K proteins accumulated in the hemolymph instead of being absorbed by the embryo. Interestingly, at the 8^th ^day of pupation, the amount of 30 K proteins decreased sharply, both in females and males. A protease for digesting 30 K proteins, named 30 kP protease A, was found in silkworm eggs, and selectively hydrolyzes the 30 kDa yolk protein of silkworm. This exhibited a high expression level in late-pupal stages, by Northern blot [19]. We predict that during this period, some 30 K proteins in male pupa are enzymatically degraded, producing nutrition for the activities of the last stages, such as eclosion and mating.

### Low molecular weight proteins change in later pupal stages

In addition to the low molecular weight lipoprotein 30 K protein, small proteins also showed remarkable variation in the silkworm hemolymph maps, especially at the 8^th ^day of pupation. Spots H52 and H53, considered pupa-specific proteins, were detected only in the 8^th ^day hemolymph of pupa, in the acidic area of the 2D map. These two proteins were identified as 32 kDa apolipoproteins. In 2005, Kim investigated a similar protein, in the fall webworm, *Hyphantrin*. The cDNA for the *Hyphantrin *protein was expressed only during the middle and late pupal stages, by Northern blot [20], and was similar to the 32 kDa apolipoprotein of silkworm in expression pattern and molecular weight. Spot H54 was more abundant at the 8^th ^day of pupation compared to the 3^rd ^day of the pupa and larval stages, and was barely detectable in the hemolymph at eclosion. This was identified as a diapause bioclock protein, EA4, which is an ATPase that measures time intervals as a diapause-duration clock. Its activity can be elevated transiently by dissociation of an inhibitory peptide under cold conditions [21]. Spots H55, H56 were identified as apolipophorin III proteins, which increased greatly in silkworm moth hemolymph. Adult moths depend on an abundance of lipids as high-powered energy sources for flight. Apolipophorin III is considered to have a key role in lipid transport systems, and serves as a reusable shuttle for transporting lipids to flight muscles, for use as fuel [22]. Although *B. mori*, as a domesticated insect, has almost lost flight capability over cultivation, many activities in the adult moth, such as mating or egg laying, still rely heavily on an efficient muscle system.

### Proteins involved in metamorphosis from larval to pupa

During insect metamorphosis, the larval tissues are degraded, and this decomposition involves programmed cell death (PCD) triggered by ecdysteroids. Some proteins related to PCD were found by 2D-electrophoresis and MS. Beta-N-acetylglucosaminidase (spots H8, H9, H10) is a chitinolytic enzyme that hydrolyzes chitose to chitobiose, and has an important role in metamorphosis [23]. The molecule 20-hydroxyecdysone induces expression of beta-N-acetylglucosaminidase, and strong expression of beta-N-acetylglucosaminidase in the latter stage of the fifth larval stage contributes to larval tissue degeneration and metamorphosis [24]. Juvenile hormone regulates growth and development in insects, and is protected from hydrolysis by general esterases by combining with juvenile hormone binding protein (JHBP) [25]. JHBP (spots H36, H37, H38) was detected in silkworm hemolymph from larval to moth stages, and showed higher expression in the larval stage. Imaginal disc growth factor (IDGF) (spots H17, H18, H19) is a polypeptide growth factor that stimulates the growth of imaginal disk cells. In the presence of 20-hydroxyecdysion, IDGF can be induced in the anterior silk gland, suggesting that IDGF is involved in PCD during silkworm metamorphosis [26].

### Proteins involved in food digestion and substance metabolism

During the fifth instar of the larval stage, the silkworm digests an abundance of mulberry to accumulate energy for the non-feeding pupal and adult stages. Some of the enzymes involved in substance and energy metabolism were found in this stage. For instants, aldose reductase (spots H21, H22) is a member of the family of oxidoreductases that convert glucose to sorbitol in carbohydrate metabolism [27]. Glyoxylate reductase (spot H23) and hydroxypyruvate isomerase (spot H31) participate in glyoxylate and dicarboxylate metabolism. The former can catalyze glycolate to produce glyoxylate, while the latter interconverts aldoses and ketoses [28,29]. Aminoacylase (spots H15, H16) belongs to a family of hydrolases that catalyze the conversion of N-acyl-L-amino acids to carboxylate and L-amino acids [30]. CI-8 (spot H12) of silkworms also showed high expression at the 6^th ^day of fifth instar. It is secreted by the fatbody during the feeding period, and sequestered in the fatbody after the onset of spinning [31]. It not only interacts with two polypetides, p29 and p60, in the midgut membrane, but also inhibits the 35-kDa protease in the digesting juice of the larval midgut [32,33]. During the period of high consumption of mulberry, we hypothesize that up-regulation of CI-8 is involved in regulating protease activity in mulberry digestion.

### Proteins related to immunity in silkworm hemolymph

Silkworm hemolymph is involved in initiation of the immune response. An abundance of proteins related to immunity were also identified in this work, including hemolin (spots H48, H49), prophenoloxidase (spot H11), serine proteinases (spots H24, H26), paralytic peptide-binding protein (H39), and trypsin inhibitor (spots H1, H13, H14). The prophenoloxidase activation system is an important defense system against parasite and pathogen invasion [34]. Some proteins of this complex system were also detected in silkworm hemolymph. Hemolin is a member of the pattern recognition proteins, which are induced after bacterial infection. Although it does not have direct antibacterial activity, it can trigger the prophenoloxidase cascade against bacterial infection, in combination with bacterial lipopolysaccharides [35]. Two serine proteases were also found in hemolymph, which shared 66% and 67% similarities to serine protease homologs (SPHs) purified from the plasma of *Manduca sexta *larvae. The SPHs of *M. sexta *are reported to bind to pattern recognition receptor and function as mediators to recruit prophenoloxidase and prophenoloxidase-activating protease to the site of infection [36]. Prophenoloxidase has long been considered a key enzyme in melanization, which is considered to be an effective defense against microorganisms. Without prophenoloxidase, the melanization of insects is almost completely inhibited [37].

### Other proteins

Some novel silkworm hemolymph proteins were identified in this work. In this work, SP1 and SP2 were found in the hemolymph of silkworm larvae, and a new hemolymph storage protein (BGIBMGA009027) was identified, which shared high similarity and a similar expression pattern to SP2. Gelsolin (spot H20) is an actin-binding protein that interacts with actin *in vitro *to promote actin nucleation and actin filament formation. There are two forms of gelsolin in vertebrates, one secreted, and other cytoplasmic [38]. The secreted gelsolin of silkworm was first reported here, in the molecular range of 45 kDa, and by hemolymph map, was expressed from larvae to moth. It is presumed to be involved in the regulation of extracellular fluid viscosity, or in wound-healing [39]. Some protease inhibitors were present in silkworm hemolymph (spot H1, H12, H13). For example, inter-alpha-trypsin inhibitors (ITIH4) are a family of structurally related serine protease inhibitors in human plasma. Recent studies suggest they may function during infection by bacteria and viruses [40]. Finally, some proteins (H2, H3, H4, H32, H51) are not discussed here, even though they had strong expression and showed remarkable change during development and metamorphosis, because of inconclusive results from database searches. Future analysis of these proteins and their functions will contribute to our understanding of development and metamorphosis.

## Conclusions

Proteomics research not only provides a method for investigating protein expression patterns, but also identifies lots of attractive candidates for further investigation. In this work, silkworm hemolymph from different stages was analyzed by proteomic tools. Some proteins, including 30 K proteins, low molecular weight lipoproteins, proteins related to PCD, proteases, protease inhibitors, enzymes involved in metabolism, proteins related to immunity, and a few novel proteins were identified from silkworm hemolymph of different stages. In general, 2D-electrophoresis of silkworm proteins during development and metamorphosis provides information for studying the proteins in silkworm hemolymph. The newly established 2D map of silkworm hemolymph may be an important tool for understanding protein function in lepidopteran insects.

## Methods

### Animals and sample preparation

The *B. mori *strain p50 (DaZao), maintained at the Key Sericultural Laboratory of Agricultural Ministry, Southwest University, was used. Silkworms were reared on mulberry at 25°C. On 1^st ^day of the fifth larval stage, male and female silkworms were determined using the following method. In females, a pair of round milky spots is located on right and left side of 8^th ^and 9^th ^abdominal segment. In males, a milky spot is located at the center of the ventral side between 8^th ^and 9^th ^abdominal segment [41]. Silkworms were pricked and squeezed gently to collect hemolymph from the 1^st ^day of fifth instar to eclosion. Each sample was collected from approximate 15 silkworms. Samples were centrifuged for 10 min at 12,000 rpm at 4°C, and stored in a lysis solution of 8 M urea, 4% (w/v) CHAPS, 1% (w/v) dithiothreitol (DTT), and 1% (v/v) protease inhibitors cocktail (Sigma P2714). Total protein content in the supernatant was determined by the method of Bradford [42].

### SDS-PAGE and 2D-PAGE

SDS-PAGE was performed using a 4% stacking gel and 12% separating gel, and 15 μg of each protein sample was loaded onto gels after dissolving in loading buffer and treating at 95°C for 5 min. SDS-PAGE was carried out at 10 mA for stacking gel and 20 mA for separating gel until the bromophenol reached the end of the gel. Resolved proteins were stained with silver nitrate. Iso-electric focusing electrophoresis (IEF) was performed with the Multiphor II system (Amersham Biosciences). About 60 μg protein was solubilized in rehydration solution (8.0 M urea, 2% CHAPS, 0.8% DTT, 0.5% IPG buffer, pH 3-10, 0.002% bromophenol blue) and applied to 13 cm pH 3-10 ReadyStrip IPG strips (Amersham). Rehydration was for 12 h at room temperature. IEF used a sequential gradient procedure of 100 V for 2 h, 200 V for 1 h, 500 V for 1 h, and 3500 V for 10 h. The current limit is 50 μA per IPGstrip. After IEF, strips were equilibrated in buffer A (50 mM Tris-HCl, pH 8.8, 6 M urea, 30% glycerol, 2% SDS and 65 mM DTT) for 15 min, then re-equilibrated in buffer B containing 2.5% iodoacetamide instead of DTT for 15 min. Strips were loaded on 12.5% polyacrylamide gels for the second-dimension separation (SE 600 Vertical Gel Electrophoresis Unit). SDS-PAGE was carried out at 5 mA per gel for 20 min, then 10 mA per gel until the bromophenol reached the end of the gel. Gels were stained with silver nitrate according to the Amersham 2D handbook and developed in rinse solution for approximate 5 min and transferred to stop solution before the background became dark [43]. At least three replicates were performed for each sample. Spots were scanned by high-resolution imagescanner II (Amersham Bioscience) at 300 pixels, and analyzed by ImageMaster 2D 5.0 software (Amersham Bioscience).

### In-gel digestion and MALDI-TOF MS

Protein spots were cut from the gel with a scalpel, and destained with 100 μL of 30 mM potassium, 100 mM sodium thiosulfate (1:1, v/v), and vortexed occasionally until the brown color disappeared completely. Pieces were rinsed three times with Milli-Q water. The supernatant was discarded and gel pieces were dehydrated with 100% ACN for 5 min, then rehydrated with 15 μL of 50 mM ammonium bicarbonate, containing 0.1 μg of modified trypsin (Sigma). After digestion overnight at 37°C, peptides were extracted three times with 25 μL of 50 mM ammonium bicarbonate containing 50% ACN containing 5% TFA. The supernatant was transferred into a new tube and the above step repeated. The combined extraction solution was concentrated to 5 μL in a vacuum centrifuge (Thermo Savant, USA). The peptide mixture (1 μL) was mixed with an equal volume of saturated CHCA solution containing 0.1% TFA and 50% ACN, and analyzed by a Voyager DE PRO MALDI-TOF MS (Applied Biosystems) using a delayed ion extraction, and positive ion reflection mode, at 20 kV of accelerating voltage, 60-65% grid voltage, and 100 ns delay time. Spectra were acquired from m/z 800 to 2500. Spectra were calibrated with trypsin auto-digestion ion peak m/z (842.510 and 2211.1046) as internal standards. MS/MS analysis was performed on a 4700 MALDI-TOF/TOF mass spectrometer (Applied Biosystems). The peak were calibrated by default and smoothed. All peaks were deisotoped.

### Database searches

Databases were constructed using "silkworm" as a key word in the NCBI protein database. Open reading frames were downloaded from silkDB http://silkworm.swu.edu.cn/silkdb/doc/download.html[44]. Combined databases containing 20,790 protein sequences were used to analyze peptide fingerprint masses using GPMAW software 6.0 [45]. The following parameters were used in all searches: maximum number of missed cleavages allowed was = 1, mass tolerance was = 1 Da, and deisotoped masses of peaks were used for protein identification. Possible covalent modifications considered in search procedure were acetylation of the N-terminus. Identification criteria were based on the number and coverage of matched peptides: minimum peptides required to match = 5, coverage of matched peptides ≥25% or the identification scores from software ≥85. In addition, the consistency of theoretical molecular weight or pI of proteins with the observed molecular weight or pI from 2D map is an important reference for mass identification.

## Abbreviations

VN: vitellogenin; SP1, SP2: storage protein 1, storage protein 2; CI: chymotrypsin inhibitor; 2D-PAGE: two-dimensional ployacrylamide gel electrophoresis; MALDI-TOF MS: matrix-assisted laser desorption ionization time of flight mass spectrometry; SDS-PAGE: sodium dodecyl sulfate polyacrylamide gel electrophoresis; CHCA: α-Cyano-4-hydroxycinnamic acid; TFA: trifluoroacetic acid; ACN: acetonitrile; PCD: programmed cell death; JHBP: juvenile hormone binding protein; IDGF: imaginal disc growth factor; SPHS: serine proteinase homologs; ITIH4: inter-alpha-trypsin inhibitor; RT-PCR: reverse transcription-PCR; PI: isoelectric point.

## Competing interests

The authors declare that they have no competing interests.

## Authors' contributions

YH and PZ conceived the idea of proteomics study, participated in its design and performed major portion of the sample analysis. YZ and XZ carried out sample preparation and 2DE analysis for silkworm hemolymph. FW and JG performed MS analysis and data analysis. QX conceived and supervised the study. All authors read and approved the final manuscript.

## Supplementary Material

Additional file 1 **Identification of the main spots from development and metamorphosis**. a. Spot number, protein name, cell function, access number in NCBI database, access number in silkworm DB database by BGI Gene Finder, number of peptides matched/total peptides, peptide coverage, score, theoretical molecular weight (Mr), theoretical pI, matched peaks and corresponding sequence are indicated. b. Some spots contained multiple proteins. Click here for file

Additional file 2 **List of 30 K proteins from 2D maps and MALDI-TOF MS**. 15 spots from 2D gels of 3^rd ^day of pupation hemolymph were excised and analyzed by MALDI-TOF MS. Five 30 K proteins were detected in silkworm hemolymph, Bmlp1, Bmlp2, Bmlp3, Bmlp4 and Bmlp7. Spot number, protein name, access number in NCBI database, access number in silkworm DB database by BGI Gene Finder, number of peptides matched/total peptides, peptide coverage, score, theoretical molecular weight (Mr), theoretical pI, matched peaks and corresponding sequence are indicated. Click here for file
