# Supplementary material for: Comparative analysis of proteome maps of silkworm hemolymph during different developmental stages
Source: Proteome Sci. 2010 Sep 8;8:45. doi: 10.1186/1477-5956-8-45 (PMC2944163; doi:10.1186/1477-5956-8-45)
Supplement: Additional file 1 — Identification of the main spots from development and metamorphosis. a. Spot number, protein name, cell function, access number in NCBI database, access number in silkworm DB database by BGI Gene Finder, number of peptides matched/total peptides, peptide coverage, score, theoretical molecular weight (Mr), theoretical pI, matched peaks and corresponding sequence are indicated. b. Some spots contained multiple proteins. [file 1477-5956-8-45-S1.DOC]

## Additional File 1: Identification of the main spots from development and metamorphosis.

| ^a^Spot No | Protein  Name; Cell Function | NCBI  entry | SilkDB  entry | No. peptides matched/  total peptides | Matched peptide coverage  (%) | Score | Mr(kDa))  /pI | Matched peaks | Corresponding  sequence |
| --- | --- | --- | --- | --- | --- | --- | --- | --- | --- |
| H1 | Inter-alpha-trypsin inhibitor heavy chain H4 precursor;  Trypsin inhibitor | No entry | BGIBMGA007558 | 10/22 | 21.9 | 114 | 84/5 | 866.391  1486.314  1682.243  1693.215  1986.113  2021.113  2167.085  2225.075  2232.081  2318.921 | YSYYDR  HIYEASDAALQLR  FVYAPDQVIESSVTK  YVVFVLDTSGSMYGR  TYYAGSEVVVAGQVDTATR  ARDSNHFTVSVNVEAFTK  YSYYDRHDVKPTLKPASK  EAAQIYQTAVSQGIGAAHIAAR  GNVEGVLGQFVVQYDVERPK  TGNEIDATKDDEQISNAEITR |
| H2 | unknown |  |  |  |  |  |  |  |  |
| H3 | unknown |  |  |  |  |  |  |  |  |
| H4 | unknown |  |  |  |  |  |  |  |  |
| H5 | SP2; Storage protein | gi|95103012 | BGIBMGA009028 | 10/19 | 17.1 | 111 | 83/5.64 | 1027.340  1278.215  1286.214  1675.033  1796.013  1812.985  1951.988  1963.894  2278.883  2466.715 | SYEVFARR  DIFIYHEGER  DPAFYQLYNR  YGIHKENDYFVYK  GEVYFYFYQQLLAR  DIFIYHEGERFPYK  RGEVYFYFYQQLLAR  IYVTKMQDGLINPEAAAK  HLNQGQFLYAFYIAVIQR  LNHSPFNVNIEVDSNVASDAVVK |
| H6 | Transferrin; Iron delivery | gi|68066547 | BGIBMGA011424 | 13/25 | 24.8 | 121 | 72/6.78 | 1015.201  1034.391  1297.229  1315.266  1459.191  1654.107  1707.998  1716.085  1844.050  1994.072  2227.851  2260.756  2393.631 | AMSVFAFSR  GHGAPELVVR  GTSFNKMEDLR  SCIVGKWSPDPK  TDLFNIYGEFLK  IPNQDFVVFQEYR  SVQDNGSDLASVDDMR  YVLHPVFHEVYGEK  KYVLHPVFHEVYGEK  IKQLADAGSSSQPEWFTK  TDEEPDAPFRYEAVIVIHK  YFILITLICACVNAAKITYK  FFGLPVGTTPASPSNENPDEYR |
| H7 | SP2; Storage protein | gi|95103012 | BGIBMGA009028 | 12/27 | 23.2 | 137 | 83/5.64 | 1278.215  1286.214  1301.248  1366.203  1675.033  1796.085  1813.058  1952.053  1963.960  2068.959  2278.976  2466.817 | DIFIYHEGER  DPAFYQLYNR  RVLGAAPMPFDK  IPEFSWYSPIK  YGIHKENDYFVYK  GEVYFYFYQQLLAR  DIFIYHEGERFPYK  RGEVYFYFYQQLLAR  IYVTKMQDGLINPEAAAK  TFVQFLQKDHFEAFGQK  VHLNQGQFLYAFYIAVIQR  LNHSPFNVNIEVDSNVASDAVVK |
| H8 | Beta-N-acetylglucosaminidase 2; Chitinolytic enzyme | gi|153791228 | BGIBMGA014116 | 14/25 | 29.3 | 163 | 61/5.34 | 919.057  1018.032  1192.869  1365.754  1407.718  1449.678  1508.584  1535.628  1599.563  1668.412  1793.334  1803.288  1810.330  1887.245 | IASPYVNR  SSSIWGILR  DIQTVIDYAR  VIPEFDVPGHTR  LGAYHETLIYTK  GNEVYEMLNILR  GEVHDFPRYPHR  LGAYHETLIYTKK  ELFHEVQALFPDR  TGGDWTEFFNKDPR  LWGHESQAAYQVYSR  DQLHINKGEVHDFPR  GLESWTHLFHLSDNR  NAPQQVLDDDTYDGPLK |
| H9 | Beta-N-acetylglucosaminidase 2; Chitinolytic enzyme | gi|153791228 | BGIBMGA014116 | 15/23 | 31.7 | 177 | 61/5.34 | 919.057  956.260  1002.300  1018.032  1193.231 1366.146  1408.129 1450.095  1508.584  1600.015 1668.882  1793.847  1810.829  1887.783  2016.760 | IASPYVNR  GEVHDFPR  DSTYTFLR  SSSIWGILR  DIQTVIDYAR  VIPEFDVPGHTR  LGAYHETLIYTK  GNEVYEMLNILR  GEVHDFPRYPHR  ELFHEVQALFPDR  TGGDWTEFFNKDPR  LWGHESQAAYQVYSR  GLESWTHLFHLSDNR  NAPQQVLDDDTYDGPLK  ASHQLIYSSGWYLDHLK |
| H10 | Beta-N-acetylglucosaminidase 2; Chitinolytic enzyme | gi|153791228 | BGIBMGA014116 | 13/26 | 28 | 167 | 61/5.34 | 919.325  956.242  1002.264  1018.288  1065.207  1193.188 1366.113  1450.047  1599.977 1668.844  1793.792  1810.815  2016.746 | IASPYVNR  GEVHDFPR  DSTYTFLR  SSSIWGILR  FDSDVLEIK  DIQTVIDYAR  VIPEFDVPGHTR  GNEVYEMLNILR  ELFHEVQALFPDR  TGGDWTEFFNKDPR  LWGHESQAAYQVYSR  GLESWTHLFHLSDNR  ASHQLIYSSGWYLDHLK |
| H11 | Prophenoloxidase;Defense and immune | gi|994749 | BGIBMGA012763 | 16/29 | 32.6 | 209 | 79/6.6 | 1087.194  1095.158  1124.109  1226.096  1365.996  1719.795  1749.684  1767.762  1789.730  1818.693  1923.677  2012.569  2125.521  2146.436  2258.481  2281.416 | EVSSVVPSGAR  LIDIFMGMR  AAIEEGYFPK  DLDRPVDQIR  SIDSSVTIPYER  WHAYIDDIFHLYK  HLEQFGVMGDSATAMR  GELFYYMHQQIIAR  LTPYGNDRLDFPNIR  FLQAIENMSVMLPNGR  RGELFYYMHQQIIAR  GEENAVFEIPDNYYPEK  GLSIPTFAESFPDKFMDPK  MPIVIPSNYTASDTEPEQR  LMIELDKFSQGVKPGNNTIR  IFIAPTVDESGKPFSFDEQR |
| H12 | CI8; Chymotrypsin inhibitor | gi|14028769 | BGIBMGA001983 | 10/21 | 32.8 | 124 | 44/5.21 | 939.362  1312.196  1320.148  1479.200  1487.218  1501.239  1853.998  1886.028  1935.108  2257.754 | AFQEAFVK  WADEQTQGHIK  SEVDNINFGDPK  MIELPYKEPGFR  GLLEDVFNLSPAGR  DIELEIPKFEIR  LLQSLFYTENEELGAK  AAEKGLLEDVFNLSPAGR  MVVVLPDKIDGLPAVLEK  EILGGGEAQESSHTYGLLNQR |
| H13 | Antitrypsin isoform 1; Trypsin inhibitor | gi|253809709 | BGIBMGA009953 | 12/27 | 32.5 | 130 | 43/5.27 | 1149.044  1181.969  1188.970  1190.988  1329.891  1589.984  1600.824  1626.963  1851.505  2003.442  2047.416  2210.294 | LDENFAVVSR  GAWSSKFDER  AIGFPDDDAIR  IFNNDAQITR  LTSDRDFYVSK  SINDWVEENTNNR  LLKGESLSVSEAIQK  DVFNSDVQNIDFSK  AIGFPDDDAIRTEFASK  YVHDGGKLDENFAVVSR  AVTNLSNVLKNGNDNFTAR  DVFNSDVQNIDFSKNTVAAK |
| H14 | Antitrypsin isoform 1; Trypsin inhibitor | gi|253809709 | BGIBMGA009953 | 11/22 | 38.8 | 124 | 43/5.27 | 1189.258  1191.274  1330.201  1590.014  1627.032  1851.938  2003.906  2210.786  2315.709  2325.679  2341.662 | AIGFPDDDAIR  IFNNDAQITR  LTSDRDFYVSK  SINDWVEENTNNR  DVFNSDVQNIDFSK  AIGFPDDDAIRTEFASK  VYVHDGGKLDENFAVVSR  DVFNSDVQNIDFSKNTVAAK  TLETAQQSMYSTEVDLYLPK  AFIEINEEGAEAAAANAFTMTR  GDYKYGESAALNAQLIEIPYK |
| H15 | Aminoacylase; Urea cycle and metabolism of amino groups | gi|87248447 | BGIBMGA011000 | 8/20 | 24.3 | 104 | 45/5.78 | 1234.353  1313.290  1390.319  1419.365  1479.209  1841.068  1904.130  2087.959 | SVGIQYIEAVR  YIIDKFMDLR  SVGIQYIEAVRR  IGLQVQVVEPLPK  SDPSVSTLQNYLR  HPPFAAEIENNVIYAR  IALNVDLKEFENMIQK  TVHLSFVPDEEIGGDTGMGK |
| H16 | Aminoacylase; Urea cycle and metabolism of amino groups | gi|87248447 | BGIBMGA011000 | 8/23 | 24.3 | 111 | 45/5.78 | 1234.301  1313.252  1390.259  1419.309  1479.148  1840.965  1904.016  2087.832 | SVGIQYIEAVR  YIIDKFMDLR  SVGIQYIEAVRR  IGLQVQVVEPLPK  SDPSVSTLQNYLR  HPPFAAEIENNVIYAR  IALNVDLKEFENMIQK  TVHLSFVPDEEIGGDTGMGK |
| H17 | Imaginal disk growth factor; Soluble polypeptide growth factor | gi|152061158 | BGIBMGA005308 | 15/29 | 35.7 | 179 | 48/8.5 | 922.530  954.329  1017.255  1076.313  1271.189  1334.162  1400.130  1530.033  1637.935  1702.973  1731.891  1917.906  1993.763  2185.669  2330.603 | ALPAVTHSK  LINPNQQK  VLCYYDSR  IVLSIATFGR  STWGSLWHGIK  YNLLLESQQAR  LVSLNENLDIDR  ESEHREGFTALVR  EADYTAPIYTPQNR  NLGGVAIVDLSLDDFR  TFGTTPVDEKESEHR  SKNLGGVAIVDLSLDDFR  NNKEADYTAPIYTPQNR  LVSLNENLDIDRTHDNYR  NPLQNADAAVTYWLTSGAPSQK |
| H18 | Imaginal disk growth factor; Soluble polypeptide growth factor | gi|152061158 | BGIBMGA005308 | 16/26 | 38.5 | 205 | 48/8.5 | 861.368  866.413  892.431  1076.382  1271.252  1334.249  1400.230  1530.153  1638.049  1703.105  1732.024  1918.022  1993.895  2185.818  2330.789  2461.565 | FGTYAFR  GMRPHLR  EGFTALVR  IVLSIATFGR  STWGSLWHGIK  YNLLLESQQAR  LVSLNENLDIDR  ESEHREGFTALVR  EADYTAPIYTPQNR  NLGGVAIVDLSLDDFR  TFGTTPVDEKESEHR  SKNLGGVAIVDLSLDDFR  NNKEADYTAPIYTPQNR  LVSLNENLDIDRTHDNYR  NPLQNADAAVTYWLTSGAPSQK  LPDDNGEGGIWVSYEDPDTAGQK |
| H19 | Imaginal disk growth factor; Soluble polypeptide growth factor | gi|152061158 | BGIBMGA005308 | 13/20 | 25.8 | 171 | 48/8.5 | 861.353  866.409  892.385  1076.387  1271.239  1334.226  1400.199  1530.113  1638.028  1703.088  1918.008  1993.873  2185.822 | FGTYAFR  GMRPHLR  EGFTALVR  IVLSIATFGR  STWGSLWHGIK  YNLLLESQQAR  LVSLNENLDIDR  ESEHREGFTALVR  EADYTAPIYTPQNR  NLGGVAIVDLSLDDFR  SKNLGGVAIVDLSLDDFR  NNKEADYTAPIYTPQNR  LVSLNENLDIDRTHDNYR |
| H20 | Gelsolin; Actin-binding protein | No entry | BGIBMGA001755 | 10/21 | 23.5 | 112 | 39/8.78 | 942.412  1005.395  1026.380  1067.399  1217.310  1249.314  1266.350  1673.076  1849.992  2212.923 | FGGAAVQHR  AISFANQVR  FFTALGSGVK  VHPAFANVGR  VLQNTEPAAFK  NYPSWVQVTR  AKVHPAFANVGR  NNLSWDIHYWIGR  AISFANQVRDQDHHGR  TATKPQEISGPITSLSDKNAR |
| H21 | Aldose reductase; Carbohydrate metabolism | No entry | BGIBMGA001351 | 12/24 | 29.4 | 132 | 37/5.37 | 845.374  883.484  948.396  1036.346  1053.448  1104.296  1488.192  1576.146  1633.148  2010.003  2113.883  2162.150 | SFDTGYR  TVPQVALR  AQPDAPPPR  YGIEDQVGR  EQVIPALRK  LWNSYHER  WSKHPDYPFQLA  AQPDAPPPRIDDER  SIGISNFNQEQIQR  LWNSYHEREQVIPALR  ENDIVVMAYTPFGSLFPSK  TVPQVALRFLIELGAVVLPK |
| H22 | Aldose reductase; Carbohydrate metabolism | No entry | BGIBMGA001351 | 13/22 | 31.2 | 158 | 37/5.37 | 845.318  883.437  925.418  948.343  1036.279  1104.236  1298.335  1386.209  1576.036  1633.042  2009.883  2113.767  2161.974 | SFDTGYR  TVPQVALR  EQVIPALR  AQPDAPPPR  YGIEDQVGR  LWNSYHER  FLIELGAVVLPK  IDDERLVSIAQK  AQPDAPPPRIDDER  SIGISNFNQEQIQR  LWNSYHEREQVIPALR  ENDIVVMAYTPFGSLFPSK  TVPQVALRFLIELGAVVLPK |
| H23 | Glyoxylate reductase; Glyoxylate and dicarboxylate metabolism | No entry | BGIBMGA009043 | 17/33 | 48.1 | 184 | 37/8.83 | 864.298  879.261  1020.247  1236.083  1331.117  1441.008  1457.046  1503.018  1682.985  1776.818  1811.835  1838.924  1860.796  2052.690  2059.845  2191.646  2463.588 | LSGFDVAR  FIYSGHR  RLSGFDVAR  RGEWEIGFDK  MKPNAVFVNVGR  YLNFGQEGSTLGR  ILEDHFTVLQSR  VLVSSNDYPPTALK  DSTVGIIGLGGIGQAVVK  GDLVDQDALYDALKNK  QIYAAGLDVTTPEPLPK\  DSTVGIIGLGGIGQAVVKR  GEWEIGFDKVLGQDLR  YLNFGQEGSTLGREEILK  LLTLPNLFVLPHIGSATVR  QIYAAGLDVTTPEPLPKDHK  VLGQDLRDSTVGIIGLGGIGQAVVK |
| H24 | Serine protease homolog 1; Prophenoloxidase-activating proteinase | gi|112983100 | BGIBMGA014404 | 9/24 | 26.7 | 109 | 46/4.82 | 875.366  898.420  1026.405  1065.303  1083.281  1418.171  2024.754  2212.743  2242.698 | NPDGVAFR  VDVPVVDR  KVDVPVVDR  TVKEIVIHK  EIYPYQDR  IRAGEWDTQNTK  DTCRGDGGSPLVCPIDYEK  AGEWDTQNTKEIYPYQDR  LGRFFQLHSTFMCAGGEPDK |
| H25 | Ester hydrolase; Hydrolases | No entry | BGIBMGA002846 | 13/20 | 36.5 | 152 | 38/6.13 | 894.321  937.272  1087.294  1103.277  1355.001  1370.976  1408.099  1608.037  1673.985  1763.977  1787.908  1791.747  2267.533 | LLEHLNR  SNFITSIR  VVGLGGAFVLR  IVSVHPVGAPK  GYFHVMPDFSR  VVGLGGAFVLRAGR  SNFITSIRETLK  LVEIGGPPYLVPQVK  TALLGNYLLTEGKPGK  LVEIGGPPYLVPQVKR  THYGDKVVGLGGAFVLR  GSSGYLQQQLPNDETR  FVEVSVADSPDLTEPPYYLK |
| H26 | Serine proteinase-like protein; Prophenoloxidase-activating proteinase | gi|114052256 | BGIBMGA009551 | 7/19 | 18 | 104 | 43/5.51 | 891.438 1128.479  1174.383  1183.370  1311.377  1699.184  2155.940 | DVFGLQGR  SLKNDVALLR  DVPAVYANVAR  IEIDMVPNPR  KIEIDMVPNPR  LVEEIIIHEDFNTK  AGEWDTQTIKEMLDHQVR |
| H27 | 30kD Bmlp1;  Low molecular lipoprotein | gi|5740 | BGIBMGA004394 | 6/9 | 27 | 74 | 29/7.63 | 1057.434  1089.411  1517.207  1626.257  1947.012  2101.838 | SYFPIQFR  FTPVLENNR  NTMDFAYQLWTK  FTPVLENNRVYFK  GSSDDRIIYGDSTADTFK  EYNSVMTLDEDMAANEDR |
| H28 | 30kD Bmlp1;  Low molecular lipoprotein | gi|5740 | BGIBMGA004394 | 7/17 | 30 | 94 | 29/7.63 | 829.479  1057.390  1064.424  1089.380  1109.357  1355.274  1946.937 | RLIENGK  SYFPIQFR  VIFTEQTVK  FTPVLENNR  LIDQQNHNK  IMSTEDKQYLK  GSSDDRIIYGDSTADTFK |
| H29 | 30kD Bmlp3;  Low molecular lipoprotein | gi|266438 | BGIBMGA004396 | 6/10 | 28.9 | 69 | 29/6.11 | 1104.349  1495.155  1594.075  1637.191  2100.818  2432.636 | AQWYLQPAK  DIVRDCFPVEFR  YDNDVLFYIYNR  LIALWENNKVYFK  YDNDVLFYIYNREYSK  DGLALTLSNDVQGDDGRPAYGDGK |
| H30 | 30kD Bmlp2;  Low molecular lipoprotein | gi|126416 | BGIBMGA004399 | 15/26 | 64.5 | 179 | 30/7.57 | 1057.385  1175.260  1192.297  1225.232  1379.215  1496.108  1592.106  1686.083  1729.094  1814.012  1916.968  1918.024  1977.870  1990.936  2105.839 | KYFPLSFR  LGSTTNPSNER  FITLWENNR  VVYGGNSADSTR  AHNTKYNQYLK  DRVVYGGNSADSTR  YENDVLFFIYNR  LYNSILTGDYDSAVR  FITLWENNRVYFK  LYNSILTGDYDSAVRK  IAYGDGVDKHTELVSWK  YFPLSFRLIMAGNYVK  EFNDALELDTIVNASGDR  NYNLALKLGSTTNPSNER  EFNDALELDTIVNASGDRK |
| H31 | Hydroxypyruvate isomerase; Glyoxylate and dicarboxylate metabolism | gi|164459610 | BGIBMGA002349 | 7/17 | 40.8 | 74 | 29/6.1 | 1020.396  1028.400  1767.105  1769.141  1817.240  2030.047  2419.929 | YFLSDYGR  GEVGVTSVPGK  TNLNTTIEYAKALDAK  AVESGFPFGFSLEQVR  LLPYIGHVQIAQVPNR  GENIQGLIEPINQYSMPK  LMLDIFHLQQIAGDITHNITK |
| H32 | unknown |  |  |  |  |  |  |  |  |
| H33 | 30kD Bmlp7;  Low molecular lipoprotein | gi|293597266 | BGIBMGA004397 | 8/16 | 29.7 | 108 | 30/8.65 | 1104.344  1134.332  1217.269  1485.120  1495.156  1623.090  1671.125  1753.007 | AQWYLQPAK  FIALWENNK  DNLFYIYNR  SEVITNVVNKLIR  DIVRDCFPVEFR  YDKDNLFYIYNR  FIALWENNKVYFK  DGLALTLSNDVHGNDGR |
| H34 | Bombyrin; Central nervous peptide | gi|112983654 | No entry | 8/20 | 32.3 | 105 | 23/7.79 | 850.402  1045.485  1122.527  1221.505  1371.437  1388.396  1397.438  1499.426 | FPNESEK  LTDDANKAAK  NVHIIDGVKK  VKNVHIIDGVK  SHQVFVWILSR  TAVDNFIKEHSK  LTVTFKFGEISR  KSHQVFVWILSR |
| H35 | Peptidylprolyl isomerase B; Protein folding | gi|95103068 | BGIBMGA004059 | 14/25 | 67.8 | 188 | 22/8.91 | 1169.218  1191.225  1457.105  1462.062  1511.139  1530.094  1597.079  1631.103  1702.993  1856.824  2085.752  2281.686  2357.627  2386.651 | FEDENFKLK  VTHKVSFDMK  DTNGSQFFITTVK  HYGAGWLSMANAGK  TPWLDGRHVVFGK  IEMTVTGANDRPVK  VIKNFMIQGGDFTK  IGDDNIGTIVIGLFGK  LKHYGAGWLSMANAGK  NFMIQGGDFTKGDGTGGR  TTENFFQLAQKPEGEGYK  DTNGSQFFITTVKTPWLDGR  TTENFFQLAQKPEGEGYKGSK  IEMTVTGANDRPVKDVVISDTK |
| H36 | Juvenile hormone binding protein; Hormone binding | gi|3098406 | BGIBMGA011549 | 6/13 | 25.9 | 65 | 27/4.89 | 1454.109  1587.126  1644.114  1784.026  1978.981  2207.907 | NQQISDFQMDTK  IAEACYISVVHNIR  MASLKVFLVFVFAR  SFTGLYTADTNVIGAVR  ADLHIVGDIVIELTEQSK  TKADLHIVGDIVIELTEQSK |
| H37 | Juvenile hormone binding protein; Hormone binding | gi|3098406 | BGIBMGA011549 | 7/17 | 33.3 | 73 | 27/4.89 | 1454.111  1587.221  1644.232  1784.342  1979.012  2208.218  2015.347 | NQQISDFQMDTK  IAEACYISVVHNIR  MASLKVFLVFVFAR  SFTGLYTADTNVIGAVR  ADLHIVGDIVIELTEQSK  TKADLHIVGDIVIELTEQSK  DSGNNSLEPDMEPLKTLR |
| H38 | Juvenile hormone binding protein; Hormone binding | gi|3098406 | BGIBMGA011549 | 7/17 | 33.3 | 73 | 27/4.89 | 1454.174  1587.163  1644.321  1784.347  1979.148  2208.341  2015.485 | NQQISDFQMDTK  IAEACYISVVHNIR  MASLKVFLVFVFAR  SFTGLYTADTNVIGAVR  ADLHIVGDIVIELTEQSK  TKADLHIVGDIVIELTEQSK  DSGNNSLEPDMEPLKTLR |
| H39^b^ | Putative paralytic peptide-binding protein homolog; Cellular immune response | No entry | BGIBMGA010876 | 15/27 | 34.9 | 163 | 50/5.46 | 905.218  1205.837  1251.753  1272.868  1280.812  1322.672  1349.661  1361.772  1389.683  1430.685  1509.561  1656.455  1784.348  1926.228  2195.049 | LWHEGHK  HTWYLYPVK  LDVNVDSYGDR  TVTISTGPITKR  DVVSQLVSHGIK  LDANVDWYGDR  LDANVDRYNDR  RHTWYLYPVK  ILNTEHEMYLK  VGDQQLFLIENR  GLLHHVDYWGEGK  GLYYGAGYELPADLR  KGLYYGAGYELPADLR  GFPQSYNVNEQFALVSK  DVVSQLVSHGIKNAMSFAYK |
|  | Putative paralytic peptide-binding protein; Cellular immune response | gi\|201023281 | BGIBMGA008165 | 11/27 | 30.9 | 132 | 48/5.53 | 1000.069  1018.338  1146.000  1295.830  1387.812  1479.723  1581.816  1605.653  1805.531  1992.370  2175.208 | TSHRVSWK  LAWGDSKDK  FIPVWENNK  LDSNVDSYNNR  ILNTEYTMYLK  QYQEVSNGPDMGR  IVTLMSAPIIPNSAR  YPYSDLPYIGQYK  VGMVVIYGMPVESQGIK  SLQDDNVGFMIEELIDR  YYNQALKLDSNVDSYNNR |
| H40 | Glycerophosphoryl diester phosphodiesterase; Glycerol metabolic process | gi|32997080 | BGIBMGA007767 | 12/27 | 33.4 | 148 | 46/5.53 | 919.042  934.968  989.004  1033.037  1188.961  1293.792  1360.844  1556.589  1657.563  1661.591  1769.299  1789.474  2396.925 | VHPYTFR  NNEVSNMK  DTDNRLGAK  TIGIYPEIK  RTIGIYPEIK  AENAFLPTEFR  LRLLQLFESDK  HDNELSLTTDVASR  YAHAAGPDKSYIIPR  LGLAMEEPVVNILHK  SNESESNSGDFEAELR  KLGLAMEEPVVNILHK  NGYRDPSAPVYIQSFEVTNLK |
| H41 | SP1;  Storage protein | gi|1335609 | BGIBMGA011266 | 24/45 | 36.2 | 273 | 88/6.95 | 929.361  1014.382  1038.369  1046.367  1059.303  1068.384  1115.430  1142.384  1158.354  1369.306  1394.277  1398.215  1414.139  1458.256  1637.205  1666.159  1694.093  1793.105  1813.062  1853.046  1860.993  1960.978  2016.960  2022.027 | DFDVFMR  FADVMIYR  EQFSFPGVK  SEDIENLAR  TSDMTFMAR  VIHLTNLMK  EGILTGKIER  FADVMIYRK  VFRVLYYAK  IRLPSGDEMPVR  EQFSFPGVKVEK  QMMDDVEMMIR  TVDAVVRIFLGPK  YTREQFSFPGVK  GEIMMYANQQLLAR  VLYYAKDFDVFMR  RLDMFELDSFMYK  RGEIMMYANQQLLAR  LLDHILQPTMFEDIK  TGMLLPTLDMTMMKDR  SSMDMQGFIPEYLSTR  GETFVHTNELQMEEAVK  SSMDMQGFIPEYLSTRR  RLNHHPFQVSIDVMSDK |
| H42^b^ | SP2; Storage protein | gi|95103012 | BGIBMGA009028 | 13/33 | 20.5 | 147 | 83/5.64 | 1139.381  1278.248  1286.247  1301.308  1366.253  1675.033  1796.050  1813.025  1929.935  1952.020  1963.954  2069.924  2156.848 | IVEYIVEFK  EDSVPMTEIMK  DPAFYQLYNR  RVLGAAPMPFDK  IPEFSWYSPIK  YGIHKENDYFVYK  GEVYFYFYQQLLAR  DIFIYHEGERFPYK  DHFEAFGQKIDFHDPK  RGEVYFYFYQQLLAR  IYVTKMQDGLINPEAAAK  TFVQFLQKDHFEAFGQK  VPNMYFKDIFIYHEGER |
|  | SP2 homolog; Storage protein | No entry | BGIBMGA009027 | 14/33 | 26.4 | 158 | 83/5.86 | 1152.380  1289.247  1300.285 1394.248  1525.286  1543.183  1794.046  1929.935  1950.041  2020.009  2038.851  2066.912  2077.986  2156.848  2324.759 | IVQYIIEFK  NLEFSIFYEK  DPAFYQLYKR  IPEFSWYSPLR  LNHKPFTVTIDIK  TFFQYLQQGHFK  GEIYFFFYQQLLAR  VGFLPKNLEFSIFYEK  RGEIYFFFYQQLLAR  QYQVPYTQEALHFVGLK  SNDYELHTEKNYEEIR  TGYLPPFNSFYYPFAQR  VAQDFNIEASKDCYTNMK  TVLILAGLIALALSSTVPEFK  SSTDFAFFKEDSLPMAEIYK |
| H43^b^ | SP2; Storage protein | gi|95103012 | BGIBMGA009028 | 15/32 | 20.3 | 185 | 83/5.64 | 1139.373  1261.257  1278.216  1286.245  1301.278  1333.222  1366.249  1398.202  1548.180  1675.077  1796.027  1813.005  1952.021  1963.926  2156.852 | IVEYIVEFK  NLEFSVFYDK  DIFIYHEGER  DPAFYQLYNR  RVLGAAPMPFDK  FFELDWFTTK  IPEFSWYSPIK  LYFDGVKITDVK  NLEFSVFYDKMR  YGIHKENDYFVYK  GEVYFYFYQQLLAR  DIFIYHEGERFPYK  RGEVYFYFYQQLLAR  IYVTKMQDGLINPEAAAK  VPNMYFKDIFIYHEGER |
|  | SP2 homolog; Storage protein | No entry | BGIBMGA009027 | 14/32 | 25.9 | 174 | 83/5.86 | 1099.350  1152.406  1289.242  1301.278  1308.339  1394.228  1525.279  1543.166  1794.058  1950.041  2038.857  2066.894  2076.940  2324.753  2356.809 | VNPGQSQITR  IVQYIIEFK  NLEFSIFYEK  DPAFYQLYKR  RIVQYIIEFK  IPEFSWYSPLR  LNHKPFTVTIDIK  TFFQYLQQGHFK  GEIYFFFYQQLLAR  RGEIYFFFYQQLLAR  SNDYELHTEKNYEEIR  TGYLPPFNSFYYPFAQR  LTNGLGKIPEFSWYSPLR  SSTDFAFFKEDSLPMAEIYK  ILSLFYNVNEISYEAEYYK |
| H44^b^ | SP2; Storage protein | gi|95103012 | BGIBMGA009028 | 13/28 | 24.8 | 144 | 83/5.64 | 1139.404  1278.288  1286.279  1300.311  1366.290  1675.141  1796.098  1813.066  1952.075  1963.986  2278.992  2324.813  2466.829 | IVEYIVEFK  EDSVPMTEIMK  DPAFYQLYNR  RVLGAAPMPFDK  IPEFSWYSPIK  YGIHKENDYFVYK  GEVYFYFYQQLLAR  DIFIYHEGERFPYK  RGEVYFYFYQQLLAR  IYVTKMQDGLINPEAAAK  VHLNQGQFLYAFYIAVIQR  ILSFFQDVSQLNTDDEYYK  LNHSPFNVNIEVDSNVASDAVVK |
|  | SP2 homolog; Storage protein | No entry | BGIBMGA009027 | 11/28 | 19.7 | 129 | 83/5.86 | 1099.398  1152.440  1 300.311  1394.281 1525.321  1543.217  1794.098  1950.085  2020.083  2038.943  2066.960  2324.813 | VNPGQSQITR  IVQYIIEFK  DPAFYQLYKR  IPEFSWYSPLR  LNHKPFTVTIDIK  TFFQYLQQGHFK  GEIYFFFYQQLLAR  RGEIYFFFYQQLLAR  QYQVPYTQEALHFVGLK  SNDYELHTEKNYEEIR  TGYLPPFNSFYYPFAQR  SSTDFAFFKEDSLPMAEIYK |
| H45 | SP1;  Storage protein | gi|1335609 | BGIBMGA011266 | 15/23 | 22 | 151 | 88/6.95 | 929.339  1014.356  1369.279  1394.251  1666.095  1694.039  1793.043  1860.917  1960.912  2016.873  2021.936  2040.947  2168.883  2252.923  2362.746 | DFDVFMR  FADVMIYR  IRLPSGDEMPVR  EQFSFPGVKVEK  VLYYAKDFDVFMR  RLDMFELDSFMYK  RGEIMMYANQQLLAR  SSMDMQGFIPEYLSTR  GETFVHTNELQMEEAVK  SSMDMQGFIPEYLSTRR  RLNHHPFQVSIDVMSDK  YYGITVTDDNLVVIDWR  YYGITVTDDNLVVIDWRK  LLDHILQPTMFEDIKEIAK  GETFVHTNELQMEEAVKVFR |
| H46^b^ | SP2; Storage protein | gi|95103012 | BGIBMGA009028 | 15/26 | 24.8 | 163 | 83/5.64 | 870.389  1027.350  1139.353  1278.226  1286.215  1366.250  1675.033  1796.032  1812.987  1929.941  1952.001  1963.910  2156.829  2278.899  2466.736 | IDFHDPK  SYEVFARR  IVEYIVEFK  EDSVPMTEIMK  DPAFYQLYNR  IPEFSWYSPIK  YGIHKENDYFVYK  GEVYFYFYQQLLAR  DIFIYHEGERFPYK  DHFEAFGQKIDFHDPK  RGEVYFYFYQQLLAR  IYVTKMQDGLINPEAAAK  VPNMYFKDIFIYHEGER  VHLNQGQFLYAFYIAVIQR  LNHSPFNVNIEVDSNVASDAVVK |
|  | SP2 homolog; Storage protein | No entry | BGIBMGA009027 | 12/26 | 20.7 | 143 | 83/5.86 | 870.389  1099.354  1152.362  1300.256  1394.226  1543.132  1794.014  1929.941  1950.022  2038.847  2066.883  2156.829 | YGAFKER  VNPGQSQITR  IVQYIIEFK  DPAFYQLYKR  IPEFSWYSPLR  TFFQYLQQGHFK  GEIYFFFYQQLLAR  VGFLPKNLEFSIFYEK  RGEIYFFFYQQLLAR  SNDYELHTEKNYEEIR  TGYLPPFNSFYYPFAQR  TVLILAGLIALALSSTVPEFK |
| H47^b^ | SP2; Storage protein | gi|95103012 | BGIBMGA009028 | 20/34 | 37.3 | 220 | 83/5.64 | 1027.349  1139.336  1278.201  1286.188  1301.236  1366.199  1398.171  1675.016  1795.983  1812.934  1951.950  1963.844  1986.887  2070.837  2156.758  2278.827  2307.685  2324.628  2356.683  2466.611 | SYEVFARR  IVEYIVEFK  DIFIYHEGER  DPAFYQLYNR  RVLGAAPMPFDK  IPEFSWYSPIK  LYFDGVKITDVK  YGIHKENDYFVYK  GEVYFYFYQQLLAR  DIFIYHEGERFPYK  RGEVYFYFYQQLLAR  IYVTKMQDGLINPEAAAK  MRDEAIALFHLFYYAK  TFVQFLQKDHFEAFGQK  VPNMYFKDIFIYHEGER  VHLNQGQFLYAFYIAVIQR  GTEGGFPFQLFVFVYPFDNK  ILSFFQDVSQLNTDDEYYK  NSNEFVIFKEDSVPMTEIMK  LNHSPFNVNIEVDSNVASDAVVK |
|  | SP2 homolog; Storage protein | No entry | BGIBMGA009027 | 15/34 | 25.9 | 186 | 83/5.86 | 1099.335  1152.332  1207.293  1289.201  1300.216  1308.306  1394.180 1525.201  1543.094  1793.955  1949.955  2038.790  2066.799  2156.758  2324.628  2356.683 | VNPGQSQITR  IVQYIIEFK  MREEAIALFK  NLEFSIFYEK  DPAFYQLYKR  RIVQYIIEFK  IPEFSWYSPLR  LNHKPFTVTIDIK  TFFQYLQQGHFK  GEIYFFFYQQLLAR  RGEIYFFFYQQLLAR  SNDYELHTEKNYEEIR  TGYLPPFNSFYYPFAQR  TVLILAGLIALALSSTVPEFK  SSTDFAFFKEDSLPMAEIYK  VYMNQGMFLYAYYIAIIQR |
| H48 | Hemolin; Insect-immune protein | gi|69146821 | BGIBMGA008736 | 9/18 | 18.8 | 106 | 44/4.98 | 939.242  1031.239  1327.052  1373.110  1394.985  1482.984  1501.076  2330.493  2486.401 | SDFGVASTR  EAPAEVLFR  TYIETPAFEEK  VTVVEGKPFELR  DGVNVDNTYKDR  RTYIETPAFEEK  KVTVVEGKPFELR  LQHTITFSVVSAPTFTTKPEK  RLQHTITFSVVSAPTFTTKPEK |
| H49 | Hemolin; Insect-immune protein | gi|69146821 | BGIBMGA008736 | 8/20 | 18 | 93 | 44/4.98 | 939.237  1031.241  1373.165  1394.932  1483.007  1501.093  2330.504  2486.419 | SDFGVASTR  EAPAEVLFR  VTVVEGKPFELR  DGVNVDNTYKDR  RTYIETPAFEEK  KVTVVEGKPFELR  LQHTITFSVVSAPTFTTKPEK  RLQHTITFSVVSAPTFTTKPEK |
| H50 | Vn; Vitellogenin | gi|871834 | BGIBMGA004585 | 16/22 | 12.5 | 244 | 203/7.16 | 1005.498  1069.410  1072.496  1134.463  1268.304  1481.412  1629.292  1753.450  1776.246  1865.269  2004.117  2026.134  2095.244  2190.106  2359.978  2432.928 | EQQQGLFR  FSFPSKDNK  TQEQQLIGR  VFEIEIDGGR  IQHVQVTCKSK  GLISALQLDTSAHR  TLAHLQEGPSSGSAFK  KIQEEEAAQVVAALPR  AETTSTVHVHPHLYGK  AEAMRPAQSILYSLSTK  LENPQHGNFNEQLPDPR  VAYHFGVPVGAEWTGTAHK  IVSLDFPTSVPVPQENLIK  YQPTPNIDKVFEIEIDGG  IIHDSQNNYDREQQQGLFR  DGVTQAGTLPAFKQIQSWIENK |
| H51 | unknown |  |  |  |  |  |  |  |  |
| H52 | 32 kDa apolipoprotein cytosolic fatty-acid binding protein; Fatty-acid binding | gi|221579621 | BGIBMGA002703 | 6/15 | 25.3 | 74 | 30/4.61 | 1142.391  1234.355 1271.311  1773.130  1774.125  2058.986 | FFTVSELGSR  LMEGSLQMIGR  FLGTWYEAER  IIVSNEIVNSLTGMK  IIVSNEIVNSLTGMKR  IIVSNEIVNSLTGMKR  RLPSLLAMQNAYAVLDR |
| H53 | 32 kDa apolipoprotein cytosolic fatty-acid binding protein; Fatty-acid binding | gi|221579621 | BGIBMGA002703 | 7/17 | 26.1 | 85 | 30/4.61 | 1142.391  1234.355  1271.311  1617.277  1773.212  2049.096  2059.062 | FFTVSELGSR  LMEGSLQMIGR  FLGTWYEAER  IIVSNEIVNSLTGMK  IIVSNEIVNSLTGMKR  LPSLLAMQNAYAVLDRFK  ERLPSLLAMQNAYAVLDR |
| H54 | Bioclock protein Diapause time regulate protein  EA4; Diapause regulation | gi|116175238 | BGIBMGA002907 | 7/17 | 58.3 | 98 | 18/6.14 | 1039.376  1160.278  1173.429  1307.345  1462.260  1956.991  2004.091 | HHGFTTPSR  DHGHPNDVNR  AIAVLSTETIR  GNITFTQVQDGK  ADDYGKSDHPDSR  HVGDLGNVVFDENHYSR  IDLVDDQISLSGPHGIIGR |
| H55 | Apoliprotein Ⅲ; Lipid transport protein | gi|112983018 | BGIBMGA013108 | 6/16 | 37.1 | 77 | 21/9.66 | 1234.346  1308.288  1543.211  1606.155  1699.060  1851.048 | VSSNVQETNEK  TLEQQFNSLTK  LQAAVQNTVQESQK  DGSESVLQQLNAFAK  DAPDFFKDIEHHTK  EFHKTLEQQFNSLTK |
| H56 | Apoliprotein Ⅲ; Lipid transport protein | gi|112983018 | BGIBMGA013108 | 9/15 | 53.2 | 108 | 21/9.66 | 1141.293  1308.243  1330.196  1421.207  1543.153  1606.099  1644.017  1699.010  1800.042 | IKAAYDDFAK  TLEQQFNSLTK  SLQGALGDANGKAK  AHPDVEKNATALR  LQAAVQNTVQESQK  DGSESVLQQLNAFAK  VSSNVQETNEKLAPK  DAPDFFKDIEHHTK  EKLQAAVQNTVQESQK |

a. Spot number, protein name, cell function, access number in NCBI database, access number in silkworm DB database by BGI Gene Finder, number of peptides matched/total peptides, peptide coverage, score, theoretical molecular weight (Mr), theoretical pI, matched peaks and corresponding sequences are indicated.

b. Some spots contained multiple proteins.
