# Supplementary material for: Comparative analysis of proteome maps of silkworm hemolymph during different developmental stages
Source: Proteome Sci. 2010 Sep 8;8:45. doi: 10.1186/1477-5956-8-45 (PMC2944163; doi:10.1186/1477-5956-8-45)
Supplement: Additional file 2 — List of 30 K proteins from 2D maps and MALDI-TOF MS. 15 spots from 2D gels of 3rd day of pupation hemolymph were excised and analyzed by MALDI-TOF MS. Five 30 K proteins were detected in silkworm hemolymph, Bmlp1, Bmlp2, Bmlp3, Bmlp4 and Bmlp7. Spot number, protein name, access number in NCBI database, access number in silkworm DB database by BGI Gene Finder, number of peptides matched/total peptides, peptide coverage, score, theoretical molecular weight (Mr), theoretical pI, matched peaks and corresponding sequence are indicated. [file 1477-5956-8-45-S2.DOC]

**Additional File 2:** List of 30K proteins from 2D maps and MALDI-TOF MS.

| Spot No | Protein name | NCBI  entry | SilkDB  entry | No. peptides matched/ total peptides | Matched peptide coverage (%) | Score | Mr(kDa)  /pI | Matched peaks | Corresponding  sequence |
| --- | --- | --- | --- | --- | --- | --- | --- | --- | --- |
| 1 | Bmlp4 | gi|126418 | BGIBMGA004395 | 10/19 | 35.4 | 137 | 30/7.58 | 1006.438  1134.456  1182.453  1192.446  1278.481  1279.471  1295.430  1592.310  1638.307  1686.299 | YFPYNFR  KYFPYNFR  LGPTLDPANER  FITLWENNR  LWVGNGQHIVR  LWVGNGQHIVR  VIFGTNTADTTR  YENDVLFFIYNR  GSIIQNVVNNLIIDK  LYNSILTGDYDSAVR |
|  | Bmlp2 | gi|156119320 | BGIBMGA004399 | 7/19 | 29.3 | 93 | 29.2/5.64 | 929.489  1057.476  1192.446  1553.417  1592.310  1686.299  1978.153 | YFPLSFR  KYFPLSFR  FITLWENNR  LIMAGNYVKLIYR  YENDVLFFIYNR  LYNSILTGDYDSAVR  EFNDALELDTIVNASGDR |
| 2 | unknown |  |  |  |  |  |  |  |  |
| 3 | unknown |  |  |  |  |  |  |  |  |
| 4 | Bmlp4 | gi|126418 | BGIBMGA004395 | 10/14 | 29.7 | 140 | 30/7.58 | 1006.283  1134.267  1182.262  1192.262  1278.277  1279.270  1295.218  1592.059  1594.042  1686.021 | YFPYNFR  KYFPYNFR  LGPTLDPANER  FITLWENNR  LWVGNGQHIVR  LWVGNGQHIVR  VIFGTNTADTTR  YENDVLFFIYNR  YENDVLFFIYNR  LYNSILTGDYDSAVR |
|  | Bmlp2 | gi|156119320 | BGIBMGA004399 | 5/14 | 17.2 | 66 | 29.2/5.64 | 1057.309  1192.262  1592.059  1594.042  1686.021 | KYFPLSFR  FITLWENNR  YENDVLFFIYNR  YENDVLFFIYNR  LYNSILTGDYDSAVR |
| 5 | Bmlp3 | gi|225905554 | BGIBMGA004396 | 9/26 | 28.5 | 97 | 29.4/6.54 | 954.358  1493.222  1495.188  1555.196  1593.166  1594.149  1594.425  2100.902  2432.731 | MAWGYNGR  DIVRDCFPVEFR  DIVRDCFPVEFR  LIFAENAIKLMYK  YDNDVLFYIYNR  YDNDVLFYIYNR  YDNDVLFYIYNR  YDNDVLFYIYNREYSK  DGLALTLSNDVQGDDGRPAYGDGK |
| 6 | Bmlp1 | gi|112984502 | BGIBMGA004394 | 10/28 | 32.4 | 126 | 29.7/9.11 | 1057.436  1089.425  1517.229  1533.220  1626.280  1627.268  1673.177  1947.044  2101.875  2103.840 | SYFPIQFR  FTPVLENNR  NTMDFAYQLWTK  VIFTEQTVKLINK  FTPVLENNRVYFK  FTPVLENNRVYFK  RNTMDFAYQLWTK  GSSDDRIIYGDSTADTFK  EYNSVMTLDEDMAANEDR  EYNSVMTLDEDMAANEDR |
| 7 | Bmlp4 | gi|126418 | BGIBMGA004395 | 9/16 | 29.7 | 130 | 30.2/7.58 | 1006.282  1134.273  1182.270  1192.264  1278.286  1279.275  1295.230  1592.069  1686.034 | YFPYNFR  KYFPYNFR  LGPTLDPANER  FITLWENNR  LWVGNGQHIVR  LWVGNGQHIVR  VIFGTNTADTTR  YENDVLFFIYNR  LYNSILTGDYDSAVR |
|  | Bmlp2 | gi|156119320 | BGIBMGA004399 | 7/16 | 29.3 | 87 | 29.2/5.64 | 929.349  1057.316  1192.264  1224.222  1592.069  1686.034  2105.810 | YFPLSFR  KYFPLSFR  FITLWENNR  VVYGGNSADSTR  YENDVLFFIYNR  LYNSILTGDYDSAVR  EFNDALELDTIVNASGDRK |
| 8 | Bmlp1 | gi|112984502 | BGIBMGA004394 | 6/22 | 35.5 | 86 | 29.7/6.11 | 1057.226  1064.283  1089.216  1109.178  1516.953  2101.487 | SYFPIQFR  VIFTEQTVK  FTPVLENNR  LIDQQNHNK  NTMDFAYQLWTK  EYNSVMTLDEDMAANEDR |
|  | Bmlp4 | gi|126418 | BGIBMGA004395 | 8/22 | 29.7 | 112 | 30.2/7.58 | 1006.200  1134.256  1182.175  1192.166  1278.184  1295.117  1591.924  1685.888 | YFPYNFR  KYFPYNFR  LGPTLDPANER  FITLWENNR  LWVGNGQHIVR  VIFGTNTADTTR  YENDVLFFIYNR  LYNSILTGDYDSAVR |
| 9 | Bmlp4 | gi|126418 | BGIBMGA004395 | 10/14 | 31.2 | 138 | 30.2/7.58 | 1006.394  1134.391  1182.383  1192.380  1278.411  1279.408  1295.354  1592.214  1686.196  1729.220 | YFPYNFR  KYFPYNFR  LGPTLDPANER  FITLWENNR  LWVGNGQHIVR  LWVGNGQHIVR  VIFGTNTADTTR  YENDVLFFIYNR  LYNSILTGDYDSAVR  FITLWENNRVYFK |
|  | Bmlp2 | gi|156119320 | BGIBMGA004399 | 6/14 | 23.4 | 77 | 29.2/5.64 | 1057.431  1192.380  1224.363  1592.214  1686.196  1729.220 | KYFPLSFR  FITLWENNR  VVYGGNSADSTR  YENDVLFFIYNR  LYNSILTGDYDSAVR  FITLWENNRVYFK |
| 10 | Bmlp4 | gi|126418 | BGIBMGA004395 | 9/18 | 25.1 | 124 | 30.2/7.58 | 1006.320  1134.320  1182.326  1192.313  1278.331  1279.321  1592.118  1594.105  1686.093 | YFPYNFR  KYFPYNFR  LGPTLDPANER  FITLWENNR  LWVGNGQHIVR  LWVGNGQHIVR  YENDVLFFIYNR  YENDVLFFIYNR  LYNSILTGDYDSAVR |
|  | Bmlp1 | gi|112984502 | BGIBMGA004394 | 6/18 | 27.0 | 74 | 29.7/6.11 | 1057.363  1089.353  1517.124  1626.184  1946.905  2101.746 | SYFPIQFR  FTPVLENNR  NTMDFAYQLWTK  FTPVLENNRVYFK  GSSDDRIIYGDSTADTFK  EYNSVMTLDEDMAANEDR |
| 11 | Bmlp1 | gi|112984502 | BGIBMGA004394 | 6/11 | 27.0 | 74 | 29.7/6.11 | 1057.435  1089.424  1517.225  1626.281  1947.025  2101.860 | SYFPIQFR  FTPVLENNR  NTMDFAYQLWTK  FTPVLENNRVYFK  GSSDDRIIYGDSTADTFK  EYNSVMTLDEDMAANEDR |
| 12 | Bmlp1 | gi|112984502 | BGIBMGA004394 | 7/13 | 28.1 | 88 | 29.7/6.11 | 1057.452  1089.445  1517.247  1626.311  1817.294  1947.060  2101.934 | SYFPIQFR  FTPVLENNR  NTMDFAYQLWTK  FTPVLENNRVYFK  NTMDFAYQLWTKDGK  GSSDDRIIYGDSTADTFK  EYNSVMTLDEDMAANEDR |
| 13 | Bmlp7 | gi|293597266 | BGIBMGA004397 | 6/15 | 21.5 | 85 | 29.3/8.65 | 954.386  1104.433  1134.415  1217.363  1623.223  1753.165 | MAWGYNGR  AQWYLQPAK  FIALWENNK  DNLFYIYNR  YDKDNLFYIYNR  DGLALTLSNDVHGNDGR |
| 14 | Bmlp7 | gi|293597266 | BGIBMGA004397 | 6/12 | 19.9 | 83 | 29.3/8.65 | 1104.441  1134.435  1217.384  1623.237  1671.296  1753.176 | AQWYLQPAK  FIALWENNK  DNLFYIYNR  YDKDNLFYIYNR  FIALWENNKVYFK  DGLALTLSNDVHGNDGR |
| 15 | Bmlp7 | gi|293597266 | BGIBMGA004397 | 5/12 | 18.0 | 72 | 29.3/8.65 | 954.312  1104.344  1217.270  1623.113  1753.039 | MAWGYNGR  AQWYLQPAK  DNLFYIYNR  YDKDNLFYIYNR  DGLALTLSNDVHGNDGR |

15 spots from 2D gels of 3^rd^ day of pupation hemolymph were excised and analyzed by MALDI-TOF MS. Five 30K proteins were detected in silkworm hemolymph, Bmlp1, Bmlp2, Bmlp3, Bmlp4 and Bmlp7. Spot number, protein name, access number in NCBI database, access number in silkworm DB database by BGI Gene Finder, number of peptides matched/total peptides, peptide coverage, score, theoretical molecular weight (Mr), theoretical pI, matched peaks and corresponding sequence are indicated.
